# Enhancing Physician-Patient Communication in Oncology Using GPT-4 Through Simplified Radiology Reports: Multicenter Quantitative Study

**DOI:** 10.2196/63786

**Published:** 2025-04-17

**Authors:** Xiongwen Yang, Yi Xiao, Di Liu, Huiyou Shi, Huiyin Deng, Jian Huang, Yun Zhang, Dan Liu, Maoli Liang, Xing Jin, Yongpan Sun, Jing Yao, XiaoJiang Zhou, Wankai Guo, Yang He, Weijuan Tang, Chuan Xu

**Affiliations:** 1 Department of Thoracic Surgery Guizhou Provincial People's Hospital Guiyang China; 2 NHC Key Laboratory of Pulmonary Immunological Diseases Guizhou Provincial People's Hospital Guiyang China; 3 Department of Cardio-Thoracic Surgery Third Affiliated Hospital of Sun Yat-sen University GuangZhou China; 4 Department of Anesthesiology Third Xiangya Hospital Central South University Changsha China; 5 Department of Thoracic Surgery Jiangxi Cancer Hospital Nanchang China; 6 Department of Pathology Guizhou Provincial People's Hospital Guiyang China; 7 Department of Medical Records and Statistics Guizhou Provincial People's Hospital Guiyang China; 8 Department of Respiratory Medicine Guizhou Provincial People's Hospital Guiyang China

**Keywords:** radiology reports, doctor-patient communication, large language models, oncology, GPT-4

## Abstract

**Background:**

Effective physician-patient communication is essential in clinical practice, especially in oncology, where radiology reports play a crucial role. These reports are often filled with technical jargon, making them challenging for patients to understand and affecting their engagement and decision-making. Large language models, such as GPT-4, offer a novel approach to simplifying these reports and potentially enhancing communication and patient outcomes.

**Objective:**

We aimed to assess the feasibility and effectiveness of using GPT-4 to simplify oncological radiology reports to improve physician-patient communication.

**Methods:**

In a retrospective study approved by the ethics review committees of multiple hospitals, 698 radiology reports for malignant tumors produced between October 2023 and December 2023 were analyzed. In total, 70 (10%) reports were selected to develop templates and scoring scales for GPT-4 to create simplified interpretative radiology reports (IRRs). Radiologists checked the consistency between the original radiology reports and the IRRs, while volunteer family members of patients, all of whom had at least a junior high school education and no medical background, assessed readability. Doctors evaluated communication efficiency through simulated consultations.

**Results:**

Transforming original radiology reports into IRRs resulted in clearer reports, with word count increasing from 818.74 to 1025.82 (*P*<.001), volunteers’ reading time decreasing from 674.86 seconds to 589.92 seconds (*P*<.001), and reading rate increasing from 72.15 words per minute to 104.70 words per minute (*P*<.001). Physician-patient communication time significantly decreased, from 1116.11 seconds to 745.30 seconds (*P*<.001), and patient comprehension scores improved from 5.51 to 7.83 (*P*<.001).

**Conclusions:**

This study demonstrates the significant potential of large language models, specifically GPT-4, to facilitate medical communication by simplifying oncological radiology reports. Simplified reports enhance patient understanding and the efficiency of doctor-patient interactions, suggesting a valuable application of artificial intelligence in clinical practice to improve patient outcomes and health care communication.

## Introduction

### Simplified Communication in Oncology

Effective physician-patient communication is essential in clinical practice, particularly in oncology, where radiology reports play a crucial role. These reports, often filled with technical jargon, can be challenging for patients to understand, impacting their engagement and decision-making.

### Large Language Models in Medical Communication

Large language models (LLMs), such as GPT-4, offer a novel approach to simplifying these reports, potentially enhancing communication and improving patient outcomes.

Previous studies have demonstrated that LLMs can effectively process and simplify complex medical texts, showing considerable potential in enhancing accessibility and comprehension for patients [1-3]. For instance, research by Amin et al [1] emphasized the role of LLMs in improving the readability of radiology reports through automated summarization, helping patients better understand their medical conditions. Similarly, studies by Doshi et al [2] focused on LLMs’ ability to streamline complex clinical information, making it more digestible for nonexpert users, particularly in the context of patient education. These studies have highlighted the improvements in readability but have largely neglected the application of LLMs in real-world clinical workflows, particularly regarding physician-patient communication and the broader impact on health care delivery, especially in oncology settings. In recent years, more research has begun to address this gap, showing that LLMs can not only enhance comprehension but also improve communication efficiency by reducing the time spent by health care providers on report interpretation and by fostering clearer interactions between physicians and patients [3-5].

### Research Gaps and Ethical Challenges

However, the application of LLMs in oncology radiology reports remains underexplored, especially regarding their potential to reduce cognitive load and streamline complex diagnostic information for better patient outcomes.

### Our Contribution

Our research aimed to fill this gap by investigating the feasibility of using GPT-4 to rewrite oncological radiology reports in a way that preserves clinical accuracy while making the content more accessible to patients. We proposed two potential workflows: (1) artificial intelligence report generation with radiologist review or (2) fully automated AI report generation. This study also examines the ethical and legal issues associated with LLMs in the medical field, providing a comprehensive assessment of their application in enhancing physician-patient communication.

By focusing on improving readability, ensuring medical accuracy, and addressing ethical considerations, our research contributes to advancing medical communication practices. The goal is to make complex medical information more accessible to patients, thereby improving their understanding and involvement in their health care.

## Methods

### Ethical Considerations

All procedures involving collection of tissues were in accordance with the ethical standards of the institutional or national research committee and with the 1964 Helsinki Declaration and its later amendments or comparable ethical standards. This retrospective compliance study was approved by the Ethics Review Committee of Guizhou Provincial People’s Hospital (2024004), the Third Affiliated Hospital of Sun Yat-sen University (B2023074), the Third Xiangya Hospital, Central South University (2024011), and Jiangxi Cancer Hospital (JC2024006). Written informed consent was obtained from individual or guardian participants. To ensure patient confidentiality, all reports were anonymized to remove any personal identifiers before analysis. This step was crucial in protecting patient rights and ensuring compliance with data privacy regulations.

### Study Design

#### Inclusion Criteria

This study included radiology reports of malignant tumors from October 2023 to December 2023, covering reports from multiple diagnostic modalities targeting the same organ (eg, computed tomography [CT] and magnetic resonance imaging, or CT and ultrasound). Histologic subtypes were confirmed using electronic medical records. The selected reports had to meet the following criteria: (1) histopathological confirmation of malignancy; (2) detailed imaging descriptions with interpretive conclusions; and (3) sufficient information to generate structured interpretative radiology reports (IRRs).

#### Exclusion Criteria

The exclusion criteria were (1) nonneoplastic lesions or benign tumors; (2) lung malignancies only receiving CT scans; (3) reports with incomplete imaging data or insufficient descriptive findings; and (4) pediatric radiology reports were excluded to maintain consistency in readability requirements, ensuring that the results were specifically applicable to adult patients.

**Figure 1:**
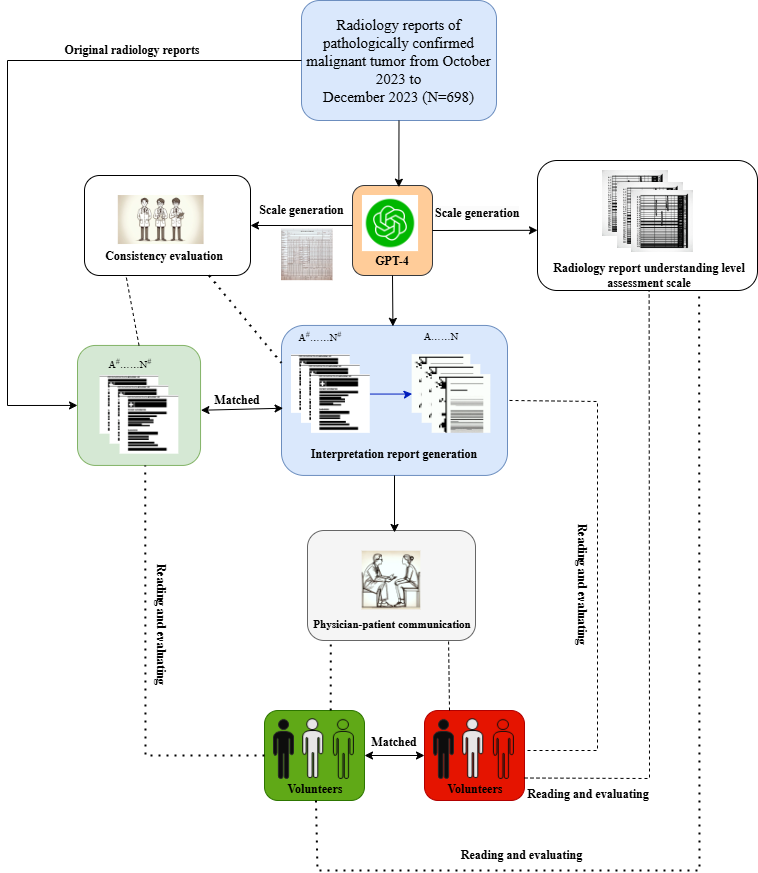
Study design flowchart. Radiology reports were processed through the GPT-4 pipeline to generate interpretive reports. Volunteers scored the reports and participated in simulated physician-patient communication. The radiological understanding scale was developed by large language models and refined by clinicians.

The design concept and process of the study are shown in Figure 1.

### Prompt Design and Parameters

To generate the IRRs, we used a standardized and structured prompt template. The primary goal of this prompt was to simplify the technical language of the reports while maintaining clinical accuracy and ensuring accessibility for nonmedical readers.

The prompt template used for GPT-4 was as follows:

Rewrite the provided radiology report into simplified, patient-friendly language, avoiding medical jargon while maintaining clinical accuracy. Ensure the report is structured with clear sections: Imaging Type, Examined Area, Major Findings, Diagnostic Explanation, and Recommendations.

Multimedia Appendix 1 clearly illustrates the transformation of the original radiology reports (ORRs) into IRRs using this prompt template. It presents both the input (ORRs) and the corresponding output (IRRs), demonstrating how complex medical language is simplified into patient-friendly language.

The reports generated by GPT-4 were reviewed and refined by 3 radiologists to ensure clinical accuracy, logical flow, and consistency with professional standards. This iterative process ensured the reports were simplified without compromising clinical relevance.

### Sample Selection and Template Development

A total of 70 (10%) radiology reports were selected from a dataset of 698 pathology reports. Reports were selected using the inclusion and exclusion criteria described in the Study Design section. The selected reports represented a variety of tumor types (eg, lung, breast, and colorectal cancer) and varied linguistic structures. Using stratified random sampling, we ensured a representative distribution of tumor types and diagnostic scenarios, which allowed for the development of generalizable templates reflective of real-world practices.

### Scale and Template Generation

**Figure 2:**
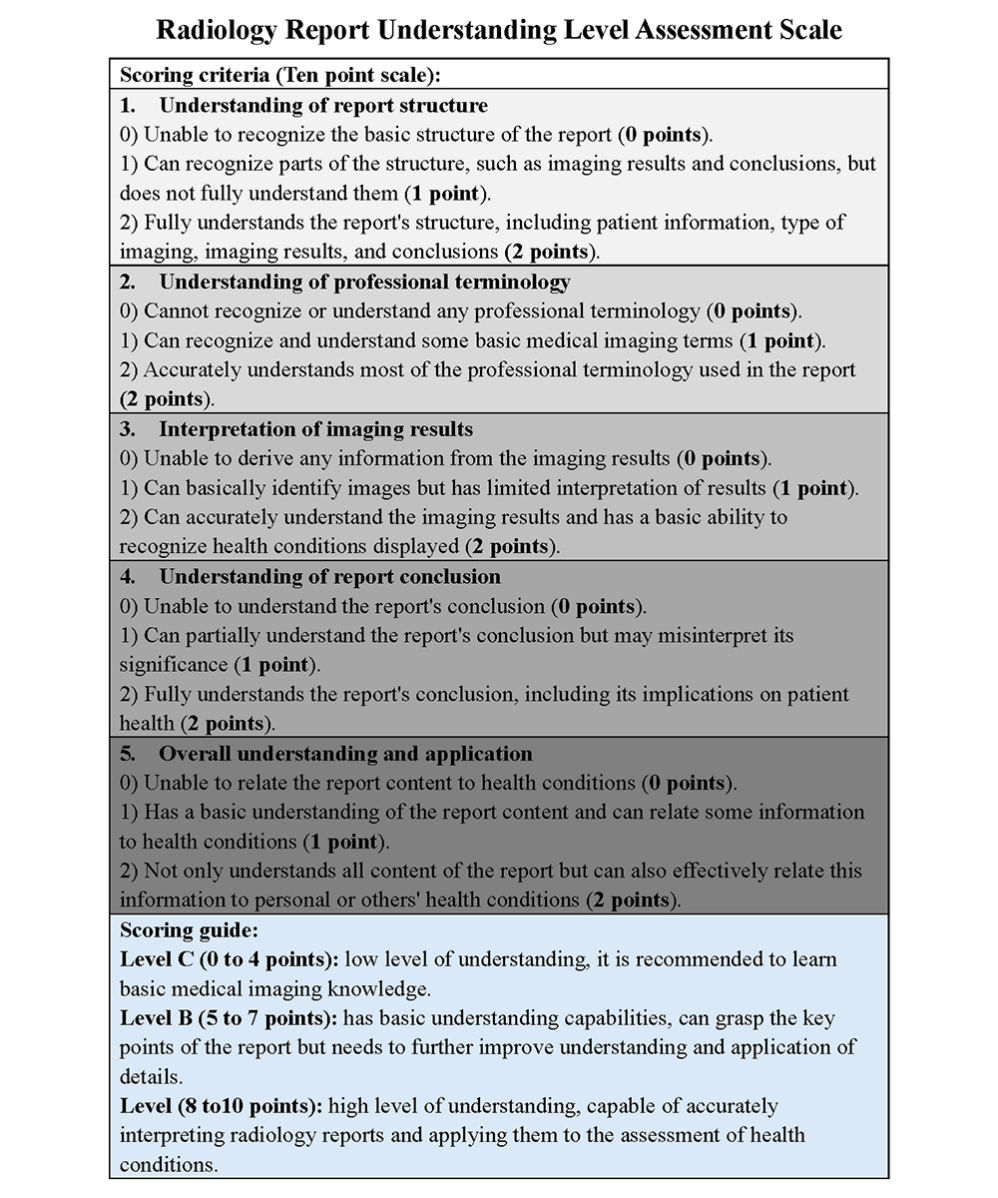
Radiology report understanding level assessment scale. This scale is designed to comprehensively assess the understanding level of individuals without a medical background regarding radiology reports, helping to identify areas of weakness and providing directions for improvement.

We created a new instrument, the Radiology Report Understanding Level Assessment Scale (Figure 2), to assess the comprehension levels of nonmedical individuals when reading radiology reports. The design of this scale was informed by foundational theories from health literacy and patient comprehension literature, emphasizing that layperson understanding of medical reports is enhanced by clear report structure, simplified terminology, accurate interpretation of imaging findings, and understanding of report conclusions [6-9]. This scale evaluated following 5 dimensions: understanding of report structure, professional terminology, imaging results, report conclusion, and overall understanding. Each dimension used a 2-point scoring system, with a maximum score of 10 points. The scale was pilot-tested to confirm interrater reliability and content validity.

To ensure the reliability and applicability of the scale, 3 experienced radiologists participated in an iterative review process, offering feedback to refine scoring criteria and ensuring that each dimension accurately captured key comprehension aspects for nonspecialist readers. The scale was subsequently pilot-tested with a subset of reports to confirm interrater reliability and content validity, yielding a high level of agreement among reviewers.

**Figure 3:**
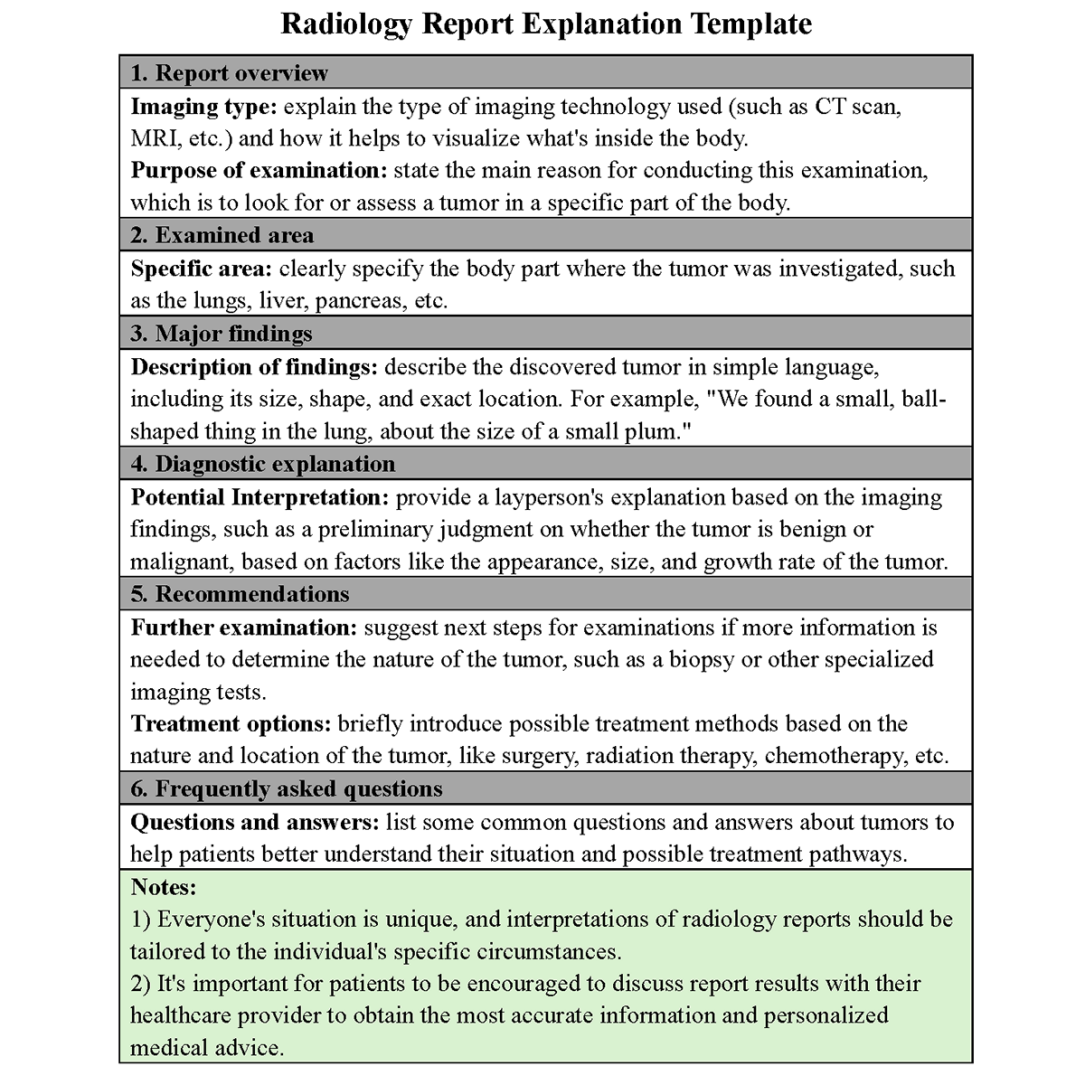
Radiology report explanation template. This template offers a basic framework to help nonprofessionals understand radiology reports related to tumors. It aims to aid patients and their families in better comprehending medical conditions, thereby facilitating more effective participation in treatment decision-making.

We developed a radiology report explanation template **(**Figure 3) as a structured framework to simplify radiology report content for nonprofessional readers. The template included sections such as imaging type, examined area, major findings, diagnostic explanation, and recommendations. These sections were designed using health literacy principles to translate technical terminology into accessible language, aiding patient comprehension [6,8,9].

In practice, GPT-4 used this template to generate IRRs from ORRs. The IRRs were then independently reviewed by 3 radiologists, who assessed each section’s readability, clinical accuracy, and relevance to ensure consistency with professional standards. This process included iterative feedback and adjustments to refine the IRRs’ clarity and applicability, providing a reliable tool for enhancing patient comprehension in oncology settings.

**Figure 4:**
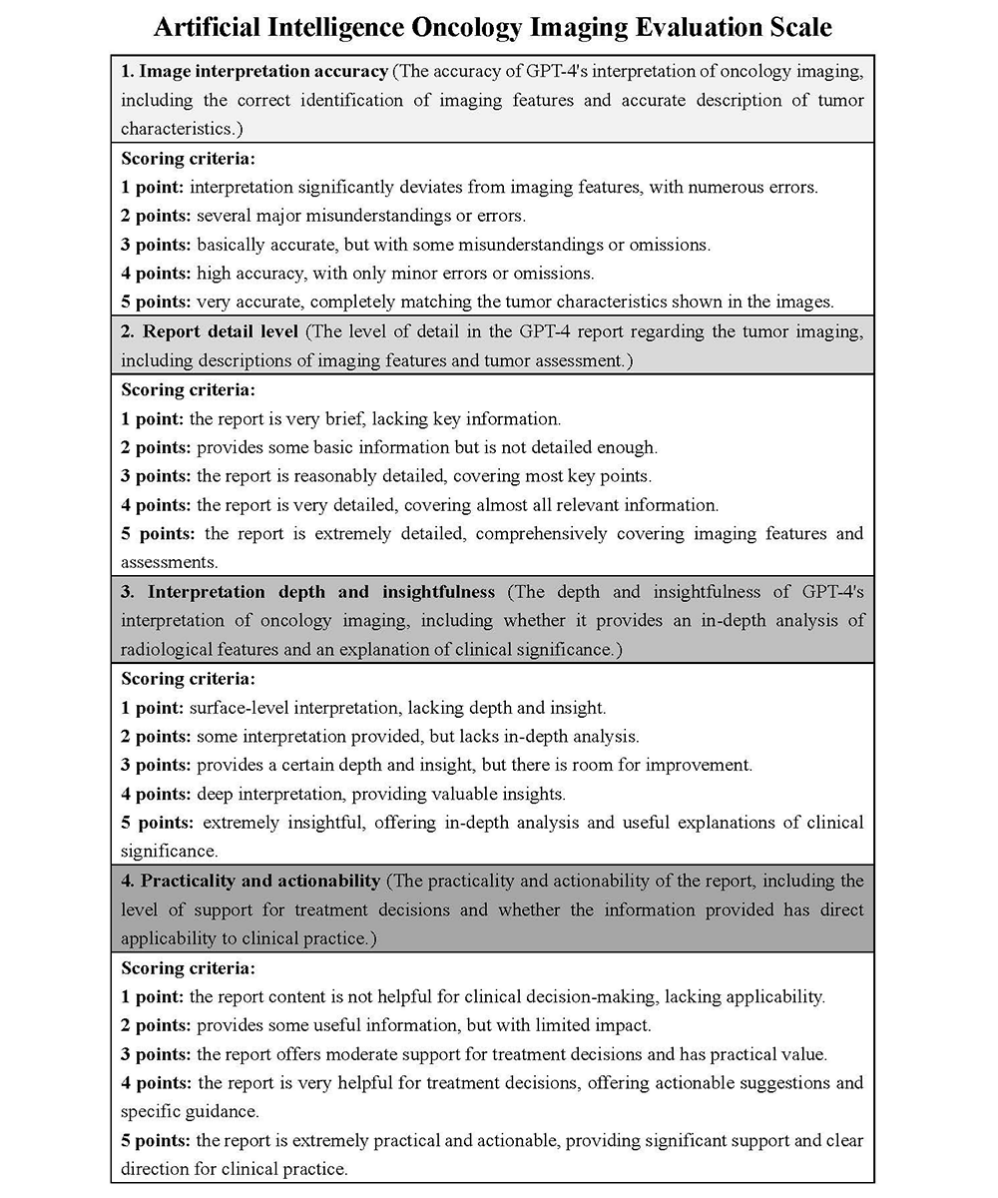
Artificial intelligence oncology imaging evaluation scale. This scale is designed to provide physicians with a structured framework for evaluating the quality of oncology imaging interpretation reports generated by GPT-4. It assesses whether artificial intelligence truly understands the content of oncology imaging reports and presents the information in a clinically valuable way.

We developed another instrument, the Artificial Intelligence Oncology Imaging Evaluation Scale (Figure 4), to assess the clinical accuracy, detail, insight, and actionability of GPT-4–generated oncology imaging reports. The design of this scale was informed by principles in diagnostic accuracy and clinical decision-making research, which emphasize the importance of accurate, detailed, and clinically relevant information for effective patient care [2,10]. The scale included 4 dimensions—image interpretation accuracy, report detail level, interpretation depth and insightfulness, and practicality and actionability—with each dimension scored on a 5-point scale to systematically assess each report’s value.

Radiologists independently reviewed each AI-generated report using the scale, with discrepancies discussed to ensure scoring consistency. An interrater reliability analysis showed a high agreement level, confirming the scale’s reliability. This rigorous process ensures that the evaluation of AI-generated reports reflects clinically meaningful criteria, enhancing the potential of these tools in real-world oncology settings.

### Participant Details

The study recruited 30 volunteers, all of whom were family members of patients, ranging in age from 20 to 67 years, with diverse educational backgrounds (junior high school to master’s degrees). None of the participants had a medical background, ensuring their perspectives closely resembled those of typical patients (Multimedia Appendix 2).

Simulated physician-patient interactions were conducted to evaluate communication efficiency. These interactions involved trained physicians explaining the radiology report findings to volunteers after they had read either the ORRs or the IRRs, with communication times meticulously recorded under controlled conditions. This set-up was designed to replicate real-world scenarios as closely as possible while adhering to ethical considerations. The evaluations were spaced 1 month apart to mitigate recall bias and ensure independent assessments of both ORRs and IRRs.

### Questionnaire Validation

The scales and questionnaires used for readability and comprehension assessment were developed based on health literacy principles and validated through a three-phase process [6,11,12]. First, 3 experienced radiologists independently reviewed the scales for clinical relevance, comprehensiveness, and clarity. Discrepancies in feedback were addressed during consensus meetings, and the instruments were iteratively refined. The finalized scales were then pilot-tested with 10 participants to confirm their applicability and appropriateness.

### Consistency and Readability Assessment

To assess the fidelity and quality of the IRRs relative to the ORRs, a GPT-4-generated consistency evaluation scale was used (Figure 4). This scale evaluated the IRRs across 4 dimensions: image interpretation accuracy, report detail level, interpretation depth and insight, and practicality and actionability. Each report was reviewed by 3 radiologists with >10 years of experience, ensuring high interrater reliability. Readability was assessed using the Chinese Readability Index Explorer 3.0, a validated tool designed to evaluate the complexity and difficulty of Chinese texts, including vocabulary, sentence structure, discourse coherence, and cognitive load [13]. This tool provided a multidimensional evaluation of the readability differences between ORRs and IRRs.

Comprehension was evaluated using the Radiology Report Understanding Level Assessment Scale (Figure 2), developed specifically for this study. The scale assessed 5 key dimensions: understanding of report structure, professional terminology, imaging results, report conclusions, and overall application to health conditions. Each dimension was scored on a 0 to 2 scale, with a maximum score of 10 points. On the basis of their total score, participants were categorized into 3 comprehension levels: level C (low comprehension, 0-4 points), level B (moderate comprehension, 5-7 points), and level A (high comprehension, 8-10 points). The number of medical terms in the ORRs and IRRs was manually counted by identifying and categorizing medical terms based on a predefined list of common medical terminology used in radiology reports [14,15]. This list was developed by consulting relevant medical dictionaries and radiology resources [15,16]. Two independent reviewers conducted the term identification process to ensure consistency and accuracy.

### Reading Time Determination

The reading time for both ORRs and IRRs was measured using a timed reading procedure. Participants were asked to read each report either aloud or silently, based on their preference. The time was recorded using a stopwatch or digital timer, starting from when the participant began reading the report until they finished. The total reading time for each report was recorded and averaged across all participants to calculate the mean reading time for both report types.

### Physician-Patient Communication

A simulated study was conducted to evaluate physician-patient communication, where volunteers were asked to read and interpret radiology reports. In the first round, the volunteers were provided with ORRs, and in the second round, they read IRRs. The volunteers were then asked to explain the patient’s condition to a physician based on what they had read.

### Statistical Analysis

Data are presented as the mean and SD. Comparisons between groups were made using ANOVA to evaluate differences between the ORRs and IRRs. The Tukey honestly significant difference post hoc test was used to identify which specific groups differed significantly from each other. This method was selected to control the type I error rate during multiple comparisons. The 95% CIs were reported for point differences to assess the precision of the findings.

The SD for key measures, such as reading time, reading rate, and comprehension scores, was calculated and presented. CIs for the point differences between ORRs and IRRs were also reported to provide additional information on the variability of the results. These changes were made to ensure that the statistical methods were more transparent and the conclusions were robust.

For measures of reliability, Cohen κ was used for categorical evaluations, and intraclass correlation coefficients (ICCs) were computed for continuous measures [17,18]. Cohen κ values and ICCs were interpreted according to established thresholds: values of 0 to 0.2 were considered poor, 0.21 to 0.4 fair, 0.41 to 0.6 moderate, 0.61 to 0.8 substantial, and 0.81 to 1 excellent.

*P* values <.05 were considered statistically significant.

## Results

### Characteristics of the Study Sample

Between October 2023 and December 2023, the study included 698 patients for analysis. The cohort consisted predominantly of female patients (408/698, 58.45%), as detailed in Table 1. The age range of the participants varied significantly, with the youngest aged 24 years and the oldest aged 82 years, resulting in an average age of 55.27 (SD 12.66) years. A substantial majority of the patients (598/698, 85.67%) were aged <65 years.

**Table 1:** Basic characteristics of patients.

Cancer sites	Age (y), mean (SD; range)	Sex, n (%)	
		Male	Female
All sites (N=698)	55.27 (12.66; 24-82)	290 (41.55)	408 (58.45)
Brain (n=32)	58.16 (11.25; 34-79)	13 (41)	19 (59)
Thyroid (n=76)	44.53 (11.75; 24-74)	32 (42)	44 (58)
Breast (n=86)	50.98 (11.32; 25-80)	0 (0)	86 (100)
Lung (n=98)	58.04 (11.64; 32-82)	49 (50)	49 (50)
Esophagus (n=10)	63.10 (7.28; 50-71)	7 (70)	3 (30)
Gastric (n=30)	55.30 (12.42; 25-80)	18 (60)	12 (40)
Liver (n=32)	61.53 (12.47; 35-82)	24 (75)	8 (25)
Pancreas (n=18)	56.39 (11.63; 37-76)	15 (83)	3 (17)
Colorectal (n=74)	61.03 (12.77; 27-82)	31 (42)	43 (58)
Kidney (n=61)	62.08 (10.02; 34-82)	31 (51)	30 (49)
Prostate (n=37)	72.89 (3.7; 67-82)	37 (100)	0 (0)
Bladder (n=50)	70.06 (5.75; 58-81)	33 (66)	17 (34)
Ovary (n=61)	52.11 (6.27; 39-68)	0 (0)	61 (100)
Uterus (n=33)	53.45 (3.24; 47-59)	0 (0)	33 (100)

### Lesion Measurement Extraction

As presented in Table 2, the mean word count of the ORRs was 818.74 (SD 197.57). Furthermore, the examination revealed an average of 17.22 (SD 4.01) medical terms within ORRs across all studied malignant tumor categories (Table 3). In addition, by using the IRR template generated by GPT-4 (Figure 3), we structured all the ORRs to maintain consistency in the output framework. It was observed that across all malignant tumors, the average word count for IRRs was 1025.82 (SD 42.87).

**Figure 5:**
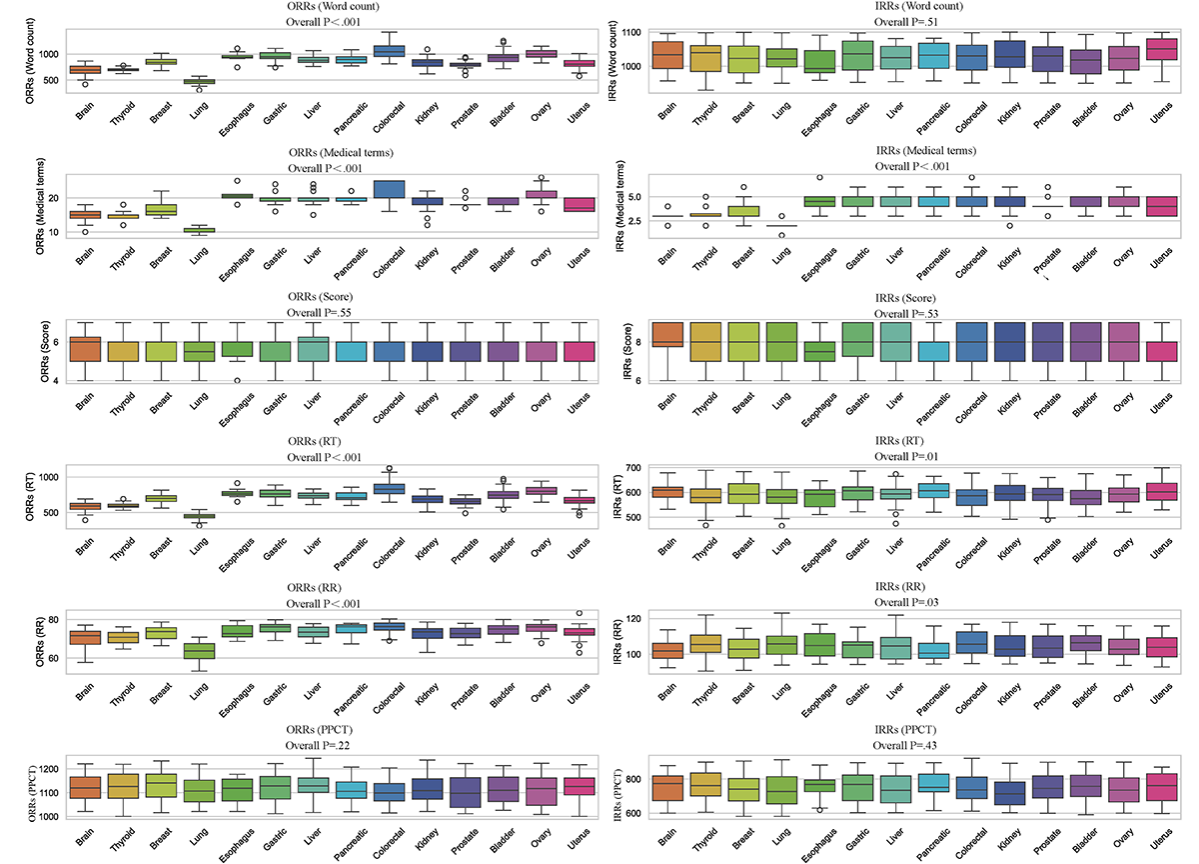
Comparative analysis of original radiology reports (ORRs) and interpretative radiology reports (IRRs) metrics across cancer sites. PPCT: physician-patient communication time; RR: reading rate; RT: reading time.

A comparison of the word count for ORRs and IRRs (Figure 5; Table 2), for all types of malignant tumors showed that the average word count for IRRs was more than that for ORRs, with this difference being statistically significant (*P*<.001).

**Table 2:** Word count of radiology reports.

Cancer sites	ORRs^a^, mean word count, n (SD; range)	IRRs^b^, mean word count, n (SD; range)	Difference (95% CI)^c^	*P* value^d^	Tukey post hoc test
All sites (N=698)	818.74 (197.57; 306-1423)	1025.82 (42.87; 930-1100)	207.08 (206.81 to 208.79)	<.001	Significant
Brain (n=32)	931.74 (119.66; 417-866)	1030.31 (44.65; 957-1096)	98.57 (88.57 to 98.31)	<.001	Significant
Thyroid (n=76)	699.72 (30.90; 625-780)	1027.8 (45.44; 930-1098)	328.08 (324.06 to 328.44)	<.001	Significant
Breast (n=86)	849.81 (70.60; 683-1015)	1022.77 (42.50; 951-1099)	172.96 (172.94 to 175.39)	<.001	Significant
Lung (n=98)	471.91 (47.96; 306-576)	1022.32 (39.28; 950-1098)	550.41 (503.55 to 507.35)	<.001	Significant
Esophagus (n=10)	948.60 (94.06; 745-1109)	1013.20 (45.61; 959-1091)	64.60 (59.55 to 72.10)	.03	Not significant
Gastric (n=30)	953.67 (96.17; 743-1109)	1033.37 (46.24; 953-1097)	79.70 (100.35 to 104.26)	<.001	Significant
Liver (n=32)	894.94 (72.03; 763-1068)	1022.28 (39.95; 955-1081)	127.34 (119.94 to 126.56)	<.001	Significant
Pancreas (n=18)	904.72 (89.65; 771-1085)	1028 (39.64; 957-1082)	123.28 (104.83 to 113.27)	<.001	Significant
Colorectal (n=74)	1065.41 (140.26; 814-1423)	1024.73 (42.27; 951-1098)	−40.68 (−73.71 to −67.91)	.01	Not significant
Kidney (n=61)	831.90 (100.53; 621-1092)	1031 (43.74; 952-1100)	199.10 (193.59 to 196.59)	<.001	Significant
Prostate (n=37)	797.81 (64.91; 593-942)	1023.54 (45.14; 951-1099)	225.73 (196.78 to 200.87)	<.001	Significant
Bladder (n=50)	685.19 (97.35; 719-1256)	1019.18 (45.12; 950-1093)	333.99 (334.28 to 342.91)	<.001	Significant
Ovary (n=61)	1009.64 (81.44; 831-1152)	1023.41 (41.94; 951-1097)	13.77 (−33.67 to 11.61)	.22	Not significant
Uterus (n=33)	810.55 (98.50; 576-1009)	1044.30 (42.54; 955-1099)	233.75 (210.98 to 218.52)	<.001	Significant

^a^ORR: original radiology report.

^b^IRR: interpretative radiology report.

^c^The 95% CI of the difference between the IRRs and ORRs.

^d^The ORRs and IRRs of different cancer sites were analyzed statistically.

**Table 3:** Number of medical terms in radiology reports.

Cancer sites	ORRs^a^, mean medical terms, n (SD; range)	IRRs^b^, mean medical terms, n (SD; range)	Difference (95% CI)^c^	*P* value^d^	Tukey post hoc test
All sites (n=698)	17.22 (4.01; 9-26)	4.2 (1; 2-7)	13.42 (12.72 to 13.32)	<.001	Significant
Brain (n=32)	14.34 (2.15; 10-18)	3.5 (0.9; 2-4)	11.31 (10.76 to 11.83)	<.001	Significant
Thyroid (n=76)	14.51 (1.35; 12-18)	3.5 (0.9; 2-5)	11.34 (10.97 to 11.53)	<.001	Significant
Breast (n=86)	16.88 (1.99; 14-22)	4.0 (1.2; 2-6)	13.17 (12.45to 13.32)	<.001	Significant
Lung (n=98)	10.26 (0.75; 9-12)	2.5 (0.6; 2-4)	8.17 (7.82to 8.84)	<.001	Significant
Esophagus (n=10)	20.9 (2.42; 18-25)	5 (1.1; 3-7)	16.30 (15.04 to 16.76)	<.001	Significant
Gastric (n=30)	19.80 (1.88; 16-24)	5 (1.2; 3-6)	15.23 (14.82 to 15.77)	<.001	Significant
Liver (n=32)	19.78 (2.11; 15-24)	4.2 (1; 3-6)	15.34 (15.14 to 16.42)	<.001	Significant
Pancreas (n=18)	20 (1.28; 18-22)	4 (1.1; 3-6)	15.61 (15.78 to 16.23)	<.001	Significant
Colorectal (n=74)	21.04 (2.74; 16-25)	4.1 (1; 2-7)	16.32 (16.30 to 17.25)	<.001	Significant
Kidney (n=61)	18.59 (2.49; 12-22)	4.2 (1.1; 2-6)	14.33 (14.97 to 15.63)	<.001	Significant
Prostate (n=37)	18.84 (1.62; 17-22)	4.1 (1.1; 3-6)	14.76 (14.72 to 15.52)	<.001	Significant
Bladder (n=50)	18.72 (1.39; 16-20)	4.2 (1.1; 2-6)	14.54 (14.24 to 14.96)	<.001	Significant
Ovary (n=61)	21.21 (2.42; 16-26)	4 (1; 2-6)	16.54 (17.00 to 17.43)	<.001	Significant
Uterus (n=33)	17.85 (1.75; 16-20)	4 (1.1; 3-6)	13.90 (13.97 to 14.57)	<.001	Significant

^a^ORR: original radiology report.

^b^IRR: interpretative radiology report.

^c^The 95% CI of the difference between the IRRs and ORRs.

^d^The ORRs and IRRs of different cancer sites were analyzed statistically.

### Consistency Evaluation of Expressed Content

The outcomes of this evaluation, as adjudicated by the 3 radiologists—identified herein as radiologist X, radiologist Y, and radiologist Z—revealed no statistical significance in their appraisals across the defined dimensions (Tables 4 and 5). Remarkably, all dimensions consistently garnered scores of 4 or above, with dimension B notably achieving a unanimous score of 5 (Table 4). This underscores the evaluative framework’s effectiveness in ensuring the IRRs’ adherence to the ORRs insights.

**Table 4:** Evaluation of consistency between original radiology reports and interpretive radiology reports for dimensions A (image interpretation accuracy) and B (report detail level).

Cancer sites		Scores for dimension A (image interpretation accuracy)						Scores for dimension B (report detail level)							
		Radiologist X	Radiologist Y	Radiologist Z	*P* value		Radiologist X		Radiologist Y		Radiologist Z		*P* value		
**All sites**						.93									—^a^
	Mean (SD)	4.49 (0.5)	4.49 (0.5)	4.48 (0.5)			5 (0)		5 (0)		5 (0)				
	Range	4-5	4-5	4-5			5-5		5-5		5-5				
**Brain**						.42								—	
	Mean (SD)	4.50 (0.51)	4.53 (0.51)	4.38 (0.49)			5 (0)		5 (0)		5 (0)				
	Range	4-5	4-5	4-5			5-5		5-5		5-5				
**Thyroid**						.79								—	
	Mean (SD)	4.45 (0.5)	4.43 (0.5)	4.49 (0.5)			5 (0)		5 (0)		5 (0)				
	Range	4-5	4-5	4-5			5-5		5-5		5-5				
**Breast**						.47								—	
	Mean (SD)	4.56 (0.5)	4.50 (0.5)	4.47 (0.5)			5 (0)		5 (0)		5 (0)				
	Range	4-5	4-5	4-5			5-5		5-5		5-5				
**Lung**						.75								—	
	Mean (SD)	4.48 (0.5)	4.49 (0.5)	4.53 (0.5)			5 (0)		5 (0)		5 (0)				
	Range	4-5	4-5	4-5			5-5		5-5		5-5				
**Esophagus**						.61								—	
	Mean (SD)	4.6 (0.5)	4.4 (0.52)	4.6 (0.52)			5 (0)		5 (0)		5 (0)				
	Range	4-5	4-5	4-5			5-5		5-5		5-5				
**Gastric**						.06								—	
	Mean (SD)	4.73 (0.45)	4.57 (0.5)	4.43 (0.5)			5 (0)		5 (0)		5 (0)				
	Range	4-5	4-5	4-5			5-5		5-5		5-5				
**Liver**						.69								—	
	Mean (SD)	4.53 (0.51)	4.44 (0.5)	4.53 (0.51)			5 (0)		5 (0)		5 (0)				
	Range	4-5	4-5	4-5			5-5		5-5		5-5				
**Pancreas**						.02								—	
	Mean (SD)	4.49 (0.5)	)4.61 (0.5)	4.22 (0.43)			5 (0)		5 (0)		5 (0)				
	Range	4-5	4-5	4-5			5-5		5-5		5-5				
**Colorectal**						.42								—	
	Mean (SD)	4.54 (0.5)	4.43 (0.5)	4.5 (0.50)			5 (0)		5 (0)		5 (0)				
	Range	4-5	4-5	4-5			5-5		5-5		5-5				
**Kidney**						.07								—	
	Mean (SD)	4.36 (0.48)	4.52 (0.5)	4.56 (0.5)			5 (0)		5 (0)		5 (0)				
	Range	4-5	4-5	4-5			5-5		5-5		5-5				
**Prostate**						.96								—	
	Mean (SD)	4.54 (0.51)	4.51 (0.51)	4.51 (0.51)			5 (0)		5 (0)		5 (0)				
	Range	4-5	4-5	4-5			5-5		5-5		5-5				
**Bladder**						.24								—	
	Mean (SD)	4.38 (0.49)	4.50 (0.51)	4.34 (0.48)			5 (0)		5 (0)		5 (0)				
	Range	4-5	4-5	4-5			5-5		5-5		5-5				
**Ovary**						.75								—	
	Mean (SD)	4.41 (0.5)	4.51 (0.5)	4.56 (0.5)			5 (0)		5 (0)		5 (0)				
	Range	4-5	4-5	4-5			5-5		5-5		5-5				
**Uterus**						.89								—	
	Mean (SD)	4.45 (0.51)	4.52 (0.51)	4.48 (0.51)			5 (0)		5 (0)		5 (0)				
	Range	4-5	4-5	4-5			5-5		5-5		5-5				

^a^Not applicable.

**Table 5:** Evaluation of consistency between original radiology reports and interpretive radiology reports for dimensions C (interpretation depth and insightfulness) and D (practicality and actionability).

Cancer sites		Dimension C (interpretation depth and insightfulness)							Dimension D (practicality and actionability)							
		Radiologist X	Radiologist Y	Radiologist Z		*P* value		Radiologist X			Radiologist Y		Radiologist Z		*P* value	
**All sites**							.91									.87
	Mean (SD)	4.49 (0.5)	4.48 (0.5)		4.47 (0.5)			4.49 (0.5)			4.5 (0.5)		4.5 (0.5)			
	Range	4-5	4-5		4-5			4-5			4-5		4-5			
**Brain**							.28									.69
	Mean (SD)	4.38 (0.49)	4.53 (0.51)		4.56 (0.5)			4.44 (0.5)			4.53 (0.51)		4.53 (0.51)			
	Range	4-5	4-5		4-5			4-5			4-5		4-5			
**Thyroid**							.23									.76
	Mean (SD)	4.43 (0.5)	4.46 (0.5)		4.57 (0.5)			4.46 (0.5)			4.51 (0.5)		4.46 (0.5)			
	Range	4-5	4-5		4-5	—^a^		4-5			4-5		4-5		—	
**Breast**							.94									.47
	Mean (SD)	4.50 (0.5)	4.48 (0.5)		4.50 (0.5)			4.51 (0.5)			4.45 (0.5)		4.55 (0.5)			
	Range	4-5	4-5		4-5			4-5			4-5		4-5			
**Lung**							.91									.95
	Mean (SD)	4.47 (0.5)	4.50 (0.5)		4.48 (0.5)			4.46 (0.5)			4.44 (0.5)		4.46 (0.5)			
	Range	4-5	4-5		4-5			4-5			4-5		4-5			
**Esophagus**							.08									.88
	Mean (SD)	4.8 (0.42)	4.3 (0.48)		4.5 (0.53)			4.3 (0.48)			4.4 (0.52)		4.4 (0.52)			
	Range	4-5	4-5		4-5			4-5			4-5		4-5			
**Gastric**							.58									.26
	Mean (SD)	4.4 (0.50)	4.47 (0.51)		4.33 (0.48)			4.47 (0.5)			4.43 (0.5)		4.63 (0.49)			
	Range	4-5	4-5		4-5			4-5			4-5		4-5			
**Liver**							.05									.85
	Mean (SD)	4.59 (0.5)	4.34 (0.48)		4.63 (0.49)			4.44 (0.51)			4.38 (0.49)		4.44 (0.5)			
	Range	4-5	4-5		4-5			4-5			4-5		4-5			
**Pancreas**							.79									.75
	Mean (SD)	4.44 (0.51)	4.33 (0.49)		4.39 (0.5)			4.39 (0.5)			4.50 (0.51)		4.50 (0.51)			
	Range	4-5	4-5		4-5			4-5			4-5		4-5			
**Colorectal**							.33									.75
	Mean (SD)	4.39 (0.49)	4.51 (0.5)		4.46 (0.5)			4.51 (0.5)			4.51 (0.5)		4.57 (0.5)			
	Range	4-5	4-5		4-5			4-5			4-5		4-5			
**Kidney**							.63									.66
	Mean (SD)	4.54 (0.5)	4.48 (0.5)		4.56 (0.5)			4.54 (0.5)			4.51 (0.5)		.46 (0.5)			
	Range	4-5	4-5		4-5			4-5			4-5		44-5			
**Prostate**							.87									.33
	Mean (SD)	4.49 (0.51)	4.43 (0.5)		4.43 (0.5)			4.60 (0.5)			4.57 (0.5)		4.43 (0.5)			
	Range	4-5	4-5		4-5			4-5			4-5		4-5			
**Bladder**							.07									.36
	Mean (SD)	4.54 (0.5)	4.48 (0.5)		4.32 (0.47)			4.40 (0.49)			4.54 (0.5)		4.50 (0.51)			
	Range	4-5	4-5		4-5			4-5			4-5		4-5			
**Ovary**							.05									.82
	Mean (SD)	4.56 (0.5)	4.50 (0.5)		4.34 (0.48)			4.62 (0.49)			4.62 (0.49)		4.57 (0.5)			
	Range	4-5	4-5		4-5			4-5			4-5		4-5			
**Uterus**							.37									.96
	Mean (SD)	4.55 (0.51)	4.67 (0.48)		.52 (0.51)			4.45 (0.51)			4.48 (0.51)		4.49 (0.51)			
	Range	4-5	4-5		44-5			4-5			4-5		4-5			

^a^Not applicable.

### Radiology Report Reading Time and Reading Rate

ORRs and IRRs were read independently by volunteers, with the two types of reports being read one month apart, and the reading time and reading rate were recorded (Figure 5; Tables 6 and 7). It was observed that the average reading time for ORRs for all types of malignant tumors was 674.86 (SD 134.08) seconds. Furthermore, for all types of malignant tumors, the average reading time for IRRs was 589.92 (SD 42.21) seconds. A comparison of the reading times for ORRs and IRRs, for all types of malignant tumors showed that the average reading time for IRRs was shorter than that for ORRs, with this difference being statistically significant (*P*<.001).

**Table 6:** Volunteers’ reading time of the original radiology reports (ORRs) and interpretative radiology reports (IRRs) generated based on GPT-4 for reading time (s).

Cancer sites	Reading time (s) for ORRs, mean (SD; range)	Reading time (s) for IRRs, mean (SD; range)	Difference (95% CI)^a^	*P* value	Tukey post hoc test
All sites	674.86 (134.08; 311-1130)	589.92 (42.21; 465-699)	84.94 (74.47 to 95.41)	<.001	Significant
Brain	584.41 (66.21; 392-691)	605.16 (32.59; 532-679)	−20.75 (−48.6 to 7.1)	<.001	Significant
Thyroid	596.14 (31.22; 531-691)	585.64 (42.95; 467-689)	10.5 (−2.53 to 23.53)	<.001	Significant
Breast	696.83 (52.65; 561-861)	596.63 (45.14; 504-684)	100.2 (85.45 to 114.95)	<.001	Significant
Lung	446.32 (41.55; 311-540)	582.54 (42.44; 465-682)	−136.26 (−147.97 to −124.54)	<.001	Significant
Esophagus	769.60 (80.88; 631-947)	580.40 (46.53; 511-647)	191.5 (131.11 to 251.89)	<.001	Significant
Gastric	758.13 (70.82; 600-888)	599.63 (37.47; 522-686)	158.50 (129.78 to 187.22)	<.001	Significant
Liver	731.66 (53.14; 612-833)	590.72 (42.39; 472-674)	140.94 (114.77 to 167.1)	<.001	Significant
Pancreas	723.39 (69.88; 599-855)	602.33 (44.92; 520-665)	121.06 (75.86 to 166.25)	<.001	Significant
Colorectal	839.24 (104.18; 642-1130)	580.11 (41.93; 504-678)	259.14 (234.18 to 284.09)	<.001	Significant
Kidney	687.66 (70.55; 508-832)	595.61 (44.90; 492-676)	92.05 (67.78 to 116.32)	<.001	Significant
Prostate	659.43 (59.08; 481-788)	589.16 (42.19; 489-660)	68.78 (45.902 to 91.67)	<.001	Significant
Bladder	746.14 (84.49; 543-974)	578.25 (37.55; 503-675)	167.84 (140.94 to 194.74)	<.001	Significant
Ovary	801.44 (60.56; 646-942)	590.67 (36.29; 520-671)	210.77 (192.77 to 228.77)	<.001	Significant
Uterus	663.42 (76.21; 459-814)	604.42 (44.58; 530-699)	59 (27.5 to 90.5)	<.001	Significant

^a^The 95% CI of the difference between the IRR and ORR word count and medical term count.

**Table 7:** Volunteers’ reading time of the original radiology reports (ORRs) and interpretative radiology reports (IRRs) generated based on GPT-4 for reading rate (words per min).

Cancer sites	Reading rate (words/min) for ORRs, mean (SD; range)	Reading rate (words/mins) for IRRs, mean (SD; range)	Difference (95% CI)^a^	*P* value	Tukey post hoc test
All sites	72.15 (5.21; 53.21-83.39)	104.70 (6.41; 90.57-123.09)	−32.55 (−33.18 to −31.93)	<.001	Significant
Brain	70.26 (5.26; 57.71-77.14)	102.36 (5.79; 92.46-113.66)	−32.10 (−34.94 to −29.26)	<.001	Significant
Thyroid	70.51 (3.03; 64.66-76.27)	105.67 (6.47; 105.07-115.27)	−35.16 (−36.86 to −33.46)	<.001	Significant
Breast	73.20 (3.29; 66.43-78.73)	103.22 (6.05; 90.98-114.55)	−30.03 (−31.39 to −28.66)	<.001	Significant
Lung	63.54 (4.57; 53.21-70.84)	105.70 (6.58; 93.92-123.09)	−42.15 (−43.72 to −40.57)	<.001	Significant
Esophagus	73.73 (3.56; 68.66-79.41)	105.23 (8.04; 94.41-116.82)	−31.50 (−38.07 to −24.93)	<.001	Significant
Gastric	75.47 (2.86; 69.10-79.87)	103.64 (5.56; 94.29-115.16)	−28.17 (−30.41 to −25.93)	<.001	Significant
Liver	73.40 (2.89; 67.67-77.95)	104.26 (7.22; 94.54-121.89)	−30.86 (−33.78 to −27.94)	<.001	Significant
Pancreas	75.08 (2.94; 67.39-78.11)	102.81 (6.71; 94.53-115.93)	−27.73 (−31.37 to −24.09)	<.001	Significant
Colorectal	76.14 (2.8; 68.97-80.39)	106.37 (6.55; 94.798-116.84)	−30.23 (−31.92 to −28.54)	<.001	Significant
Kidney	72.52 (3.72; 62.93-78.75)	104.25 (6.27; 94.54-117.92)	−31.72 (−33.72 to −29.72)	<.001	Significant
Prostate	72.84 (3.05; 66.76-78.10)	104.6 (6.5; 95.16-116.80)	−31.76 (−34.14 to −29.38)	<.001	Significant
Bladder	74.86 (3.12; 68.16-80.02)	106 (5.65; 94.61-116.04)	−31.14 (−32.99 to −29.29)	<.001	Significant
Ovary	75.60 (2.70; 67.71-79.82)	104.25 (6.3; 93.80-115.95)	−28.65 (−30.40 to −26.89)	<.001	Significant
Uterus	73.34 (3.61; 62.74-83.39)	104.04 (6.45; 92.88-115.85)	−30.70 (−33.24 to −28.16)	<.001	Significant

^a^The 95% CI of the difference between the IRRs and ORRs.

It was observed that the average reading time for ORRs for all types of malignant tumors was 72.15 words per minute. Furthermore, for all types of malignant tumors, the average reading time for IRRs was 104.70 words per minute. A comparison of the reading rates for ORRs and IRRs, for all types of malignant tumors showed that the average reading rates for IRRs was faster than that for ORRs, with this difference being statistically significant (*P*<.001).

### Readability Assessment

The readability of ORRs and IRRs was assessed using the Chinese Readability Index Explorer 3.0. Detailed results are provided in Multimedia Appendix 3. The key findings of the assessment were as follows: for vocabulary, the advanced vocabulary ratio decreased from 18.5% (ORRs) to 3.2% (IRRs). The Lexical Diversity Index reduced from 0.75 in ORRs to 0.45 in IRRs. For sentence structure, the average sentence length was reduced from 32 words (ORRs) to 18 words (IRRs). The complex sentence ratio dropped from 45% in ORRs to 12% in IRRs. For discourse coherence, the coherence score improved from 70 (ORRs) to 85 (IRRs). The average paragraph length decreased from 220 words (ORRs) to 140 words (IRRs). For cognitive load, the information density index was reduced from 0.72 (ORRs) to 0.45 (IRRs). The complex vocabulary density decreased from 18.5% (ORRs) to 4% (IRRs).

### Understanding Level Assessment

Comprehension of ORRs and IRRs showed significant improvements across all evaluated dimensions (Figure 2; Table 8).

**Table 8:** Volunteers’ evaluation of the original radiology reports (ORRs) and interpretative radiology reports (IRRs) generated based on GPT-4 for comprehension score.

Cancer sites	Comprehension score for ORRs, mean (SD; range)	Comprehension score for IRRs, mean (SD; range)	Difference (95% CI)^a^	*P* value	Tukey post hoc test
All sites	5.51 (0.89; 4-7)	7.83 (0.99; 6-9)	−2.32 (−2.40 to −2.24)	<.001	Significant
Brain	5.59 (1.07; 4-7)	8.13 (0.87; 6-9)	−2.53 (−2.97 to −2.09)	<.001	Significant
Thyroid	5.45 (0.85; 4-7)	7.8 (1.05; 6-9)	−2.36 (−2.62 to −2.09)	<.001	Significant
Breast	5.38 (0.86; 4-7)	7.74 (0.95; 6-9)	−2.36 (−2.58 to −2.14)	<.001	Significant
Lung	5.46 (0.89; 4-7)	7.81 (1.05; 6-9)	−2.35 (−2.56 to −2.13)	<.001	Significant
Esophagus	5.7 (0.67; 4-7)	7.4 (0.97; 6-9)	−1.70 (−2.29 to −1.11)	<.001	Significant
Gastric	5.87 (0.78; 4-7)	8.06 (0.87; 6-9)	−2.20 (−2.60 to −1.80)	<.001	Significant
Liver	5.75 (0.98; 4-7)	7.94 (1.08; 6-9)	−2.19 (−2.59 to −1.78)	<.001	Significant
Pancreas	5.44 (0.7; 4-7)	7.56 (0.98; 6-9)	−2.11 (−2.56 to −1.66)	<.001	Significant
Colorectal	5.51 (0.86; 4-7)	7.74 (0.98; 6-9)	−2.23 (−2.48 to −1.98)	<.001	Significant
Kidney	5.51 (0.94; 4-7)	7.9 (1.01; 6-9)	−2.39 (−2.65 to −2.13)	<.001	Significant
Prostate	5.49 (1.02; 4-7)	7.84 (1.07; 6-9)	−2.35 (−2.78 to −1.93)	<.001	Significant
Bladder	5.58 (0.88; 4-7)	8 (0.9; 6-9)	−2.42 (−2.79 to −2.05)	<.001	Significant
Ovary	5.39 (0.82; 4-7)	7.72 (1.03; 6-9)	−2.35 (−2.56 to −2.13)	<.001	Significant
Uterus	5.52 (0.94; 4-7)	7.79 (0.96; 6-9)	−2.27 (−2.58 to −1.96)	<.001	Significant

^a^The 95% CI of the difference between the IRRs and ORRs.

The average communication time for ORRs across all cancer types was 1116.11 (SD 57.87) seconds, whereas for IRRs it was significantly reduced to 745.30 (SD 85.08) seconds (*P*<.001). These results indicate that IRRs not only enhance patient understanding but also streamline the communication process, making it more time efficient. The simulated physician-patient communication workflow demonstrated significant differences in communication efficiency between ORRs and IRRs.

The understanding of report structure score increased from 0.8 (SD 0.4) ORRs to 1.8 (SD 0.2) IRRs (*P*<.001). The understanding of professional terminology score rose from 0.7 (SD 0.3) ORRs to 1.7 (SD 0.3) IRRs (*P*<.001). The interpretation of imaging results score improved from 0.6 (SD 0.5) ORRs to 1.7 (SD 0.2) IRRs (*P*<.001). The understanding of report conclusion score increased from 0.9 (SD 0.3) ORRs to 1.9 (SD 0.1) IRRs (*P*<.001). Similarly, the overall understanding and application score rose from 0.9 (SD 0.4) ORRs to 1.8 (SD 0.2) IRRs (*P*<.001). The total comprehension score increased significantly from 5.51 (SD 0.89) ORRs to 7.83 (SD 0.99) IRRs (*P*<.001). Detailed results are provided in Table 8.

To assess the reliability of these evaluations, Cohen κ values were estimated and they ranged from 0.76 to 0.85 for categorical evaluations, indicating substantial interrater agreement. For continuous measures, ICCs ranged from 0.82 to 0.91 across comprehension dimensions, reflecting excellent interrater reliability (Multimedia Appendices 4 and 5).

### Physician-Patient Communication

After volunteers finished reading the ORRs, the physician engaged in simulated physician-patient communication with the volunteers to explain the patient’s condition and recorded the communication time (Figure 5; Table 9). Across all malignant tumors, the average communication time was 1116.11 (SD 57.87) seconds. In addition, after volunteers finished reading the IRRs, the physician conducted simulated physician-patient communication based on the report content, explained the patient’s condition, and recorded the communication time. Across all malignant tumors, the average communication time was 745.30 (SD 85.08) seconds. Further analysis revealed that, regardless of the type of malignant tumor, the communication time following the reading of the ORRs was significantly longer than the time following the reading of IRRs (*P*<.001).

**Table 9:** Volunteers’ evaluation of the original radiology reports (ORRs) and interpretative radiology reports (IRRs) generated based on GPT-4 for physician-patient communication time (s).

Cancer sites	Communication time (s) for ORRs, mean (SD; range)	Communication time (s) for IRRs, mean (SD; range)	Difference (95% CI)^a^	*P* value	Tukey post hoc test
All sites	1116.11 (57.87; 1001-1237)	745.30 (85.08; 582-921)	370.81 (363.14-378.48)	<.001	Significant
Brain	1116.59 (56.39; 1021-1221)	752.47 (81.78; 601-878)	364.13 (332.19-396.06)	<.001	Significant
Thyroid	1125.22 (59.48; 1001-1219)	762.75 (86.29; 606-899)	362.47 (338.91-386.04)	<.001	Significant
Breast	1130.60 (57.74; 1016-1233)	739.88 (84.84; 601-905)	390.72 (369.17-412.27)	<.001	Significant
Lung	1110.10 (56.74; 1021-1220)	735.07 (91.55; 582-913)	375.03 (354.07-395.99)	<.001	Significant
Esophagus	1112.70 (56.45; 1021-1189)	755.20 (83.20; 619-882)	357.50 (268.84-446.16)	<.001	Significant
Gastric	1121.63 (58.69; 1012-1222)	751.60 (93.86; 611-896)	370.03 (328.36-411.70)	<.001	Significant
Liver	1125.19 (52.58; 1017-1244)	742.37 (89.41; 603-892)	382.81 (341.45-424.18)	<.001	Significant
Pancreas	1108.39 (52.43; 1020-1207)	766.11 (81.23; 615-895)	342.28 (289.21-395.35)	<.001	Significant
Colorectal	1105.11 (53.28; 1015-1204)	747.42 (79.38; 615-921)	357.69 (334.74-380.64)	<.001	Significant
Kidney	1112.88 (57.38; 1021-1237)	720.48 (84.71; 604-892)	392.41 (364.15-420.67)	<.001	Significant
Prostate	1100.76 (68.76; 1014-1222)	752.86 (77.51; 600-898)	347.89 (314.36-381.42)	<.001	Significant
Bladder	1112.94 (57.31; 1026-1208)	755.64 (83.34; 591-900)	357.30 (328.70-385.90)	<.001	Significant
Ovary	1112.26 (61.53; 1009-1223)	740.39 (84.06; 601-899)	371.87 (346.16-397.58)	<.001	Significant
Uterus	1125.91 (54.52; 1001-1224)	751.45 (81.08; 599-870)	374.45 (339.56-409.35)	<.001	Significant

^a^The 95% CI of the difference between the IRRs and ORRs.

## Discussion

### Principal Findings

AI has influenced every aspect of medical practice [19-22], with recent applications of LLMs further enhancing clinical diagnostic and treatment processes [2,3,10,23,24]. This study demonstrates the considerable potential of AI in improving the understandability of medical information by simplifying oncological radiology reports using GPT-4. Our research confirms that LLMs can effectively transform complex radiology reports into formats that are more accessible to patients, significantly improving both the efficiency of physician-patient communication and patient comprehension. Specifically, we found that IRRs generated by GPT-4 reduced communication time between physicians and patients while enhancing patients’ understanding of the report content.

Our study extends existing research by not only simplifying radiology reports but also standardizing and structuring the IRRs through the use of GPT-4 [1]. In addition, the diverse characteristics of the 30 volunteers, ranging in age from 20 to 67 years and with educational backgrounds from junior high school to master’s degrees, ensured that the reports were accessible to a wide demographic. Despite an increase in word count, reading rates and comprehension significantly improved. To minimize bias, the evaluations of ORRs and IRRs were conducted 1 month apart.

We observed that in simulated physician-patient interactions, the use of IRRs led to shorter interactions and improved patient comprehension. Although a 1-month temporal separation between the 2 rounds was introduced to minimize potential learning effects, it is possible that some residual influence from the first round of explanations persisted. However, we believe that the impact of this effect was minimal and does not compromise the validity of the study’s findings [25].

We also acknowledge the potential for bias due to the lack of blinding, as both physicians and volunteers were aware of whether they were using ORRs or IRRs [26,27]. To mitigate this, physicians adhered to a standardized script to ensure consistency in explanations, and volunteers were kept unaware of the study’s hypotheses. In addition, objective metrics, such as reading time and comprehension scores, were used to provide an unbiased and reliable evaluation of the communication process, further strengthening the robustness of our conclusions [26,27].

For future studies, we plan to implement stronger blinding techniques and explore crossover designs to minimize bias further. In addition, the integration of LLMs in health care introduces important ethical and legal considerations [28-32]. Issues surrounding patient data privacy, AI bias, and the legal implications of AI-generated medical reports must be addressed. Patient data used to train AI systems must be handled securely, adhering to privacy laws, such as Health Insurance Portability and Accountability Act [29]. Moreover, biased datasets could lead to inaccurate medical recommendations, highlighting the need for diverse, representative datasets [30]. The legal ramifications of using AI-generated reports are also critical, as errors in diagnosis or treatment could lead to malpractice claims [31,32]. Therefore, health care providers must remain responsible for final medical decisions, ensuring that AI tools complement, rather than replace, clinical judgment [32].

### Limitations

This study has several limitations. First, most radiology report conclusions are negative, and this study only included malignant tumor reports, limiting its applicability to negative findings. Nonetheless, we believe that with appropriate adjustments, GPT-4 has the potential to improve communication efficiency even for reports with negative findings. Second, while GPT-4 generated reports currently require review by radiologists or clinicians, the time needed for such reviews is significantly less than that required for direct physician-patient communication. As the model continues to iterate, its accuracy is expected to improve, further reducing the workload for health care providers. Third, although the standardized prompt effectively simplified the reports for a diverse group of nonmedical readers, we acknowledge that a uniform approach may not fully address the needs of individuals with varying educational or cultural backgrounds. Future research should explore personalized prompt adjustments to generate reports tailored to specific patient or family member characteristics. Fourth, as discussed in the Introduction section, this study focused exclusively on radiology reports related to malignant tumors. While the findings provide important insights into the accuracy and applicability of GPT-4 for analyzing these specific reports, it is important to recognize that the model’s performance may vary in different contexts. For example, GPT-4 might perform differently in reports involving negative or benign findings, as the model’s training and optimization are heavily influenced by the presence of clinically significant conditions, such as malignant tumors. The language, terminology, and diagnostic challenges associated with benign findings are distinct from those related to malignancies, which could affect the model’s ability to detect and interpret subtle or less critical abnormalities. In addition, the prevalence of normal findings in these reports may introduce different linguistic patterns that GPT-4 might handle with less accuracy, depending on its exposure to such data during training. Therefore, it is essential to explore how GPT-4 can be adapted to handle a broader range of radiology reports, including those with negative or benign findings, in future studies. This would provide a more comprehensive understanding of the model’s potential applications across diverse diagnostic contexts.

Looking ahead, future studies should aim to develop more advanced models that account for the complexity and nuances of medical reports. In addition, it is crucial to assess patient trust and acceptance of AI-generated reports. Another promising direction is leveraging LLMs for personalized medical information delivery, tailoring content to patients’ unique backgrounds and preferences to further enhance comprehension, engagement, and satisfaction.

### Conclusions

Our study underscores the potential value of using LLMs to simplify radiology reports in enhancing physician-patient communication efficiency and patient understanding. As we further explore the application of these technologies, we anticipate not only ensuring the quality and efficiency of medical care but also better meeting the personalized needs of patients, fostering continuous innovation and development in the medical field.

## Appendix

Multimedia Appendix 1 Example of original radiology report and corresponding interpretative radiology report.

Multimedia Appendix 2 Demographic characteristics of volunteers.

Multimedia Appendix 3 Chinese Readability Index Explorer 3.0 Readability Metrics Comparison: original radiology reports versus interpretative radiology reports.

Multimedia Appendix 4 Cohen κ for categorical evaluations of comprehension dimensions.

Multimedia Appendix 5 Intraclass correlation coefficients for continuous evaluations of comprehension dimensions.

Multimedia Appendix 6 Representative GPT-4 interactions used during manuscript preparation.

## Abbreviations

AI: artificial intelligence
CT: computed tomography
ICC: intraclass correlation coefficient
IRR: interpretative radiology report
LLM: large language model
ORR: original radiology report

## Data Availability

The datasets generated and analyzed during this study are not publicly available due to institutional data protection policies but are available from the corresponding author on reasonable request.
