# Supplementary material for: Enhancing Physician-Patient Communication in Oncology Using GPT-4 Through Simplified Radiology Reports: Multicenter Quantitative Study
Source: J Med Internet Res. 2025 Apr 17;27:e63786. doi: 10.2196/63786 (PMC12046253; doi:10.2196/63786)
Supplement: Multimedia Appendix 1 [file jmir_v27i1e63786_app1.docx]

**Table S1.** Example of Original Radiology Report and Corresponding Interpretive Radiology Report.

| Original Radiology Report | Interpretive Radiology Report |
| --- | --- |
| Examination Site: Pulmonary High-Resolution CT Scan with 3D Imaging | Examination Site: Pulmonary High-Resolution CT Scan with 3D Imaging |
| **Findings:**  **Right Upper Lobe (Posterior Segment):** A nodule with mixed ground-glass opacity measuring approximately 11mm x 7mm is observed, with a lobulated shape and spiculated borders.  **Left Lung Oblique Fissure:** A small nodule with increased density, approximately 5mm x 3mm in size, is seen.  **Both Lungs:** Increased and disordered bronchovascular bundles are observed. Scattered patchy and linear areas of increased density are noted in both lungs. The anterior segment of the right upper lobe and parts of the bilateral lower lobes show a reticular pattern.  **Left Lower Lobe (Outer Basal Segment):** A round translucent area approximately 5mm in diameter is seen beneath the pleura, with surrounding patchy areas of increased density.  **Mediastinum: Positioned centrally.** The trachea, bronchi, and their major branches are patent.  **Heart:** Size and shape are normal, with no abnormalities detected.  **Aorta:** A nodular high-density area is visible on the aortic wall, consistent with atherosclerosis.  **Pleural Effusion:** No pleural effusion is observed in either lung.  **Chest Wall:** Bilateral chest walls are symmetric, and the bone structure of the chest is intact.  **Liver:** A slightly hypodense area in the left lobe of the liver, approximately 20mm x 20mm, is noted. Further abdominal examination is recommended. | **Findings:**  **Right Upper Lung (Posterior Segment):** A nodule about 11mm x 7mm is seen in the upper part of the right lung. It has an irregular shape with some rough edges, which may suggest the possibility of a new growth. Further testing is recommended to confirm this.  **Left Lung Oblique Fissure:** A small nodule, approximately 5mm x 3mm in size, is observed in the left lung. It is not immediately concerning but should be monitored over time with follow-up scans.  **Both Lungs:** There are scattered areas of increased density in both lungs, which could be due to long-standing inflammation or scarring from previous infections. Some areas in the right upper lobe and both lower lungs show a mesh-like pattern, which might suggest ongoing changes in the lung tissue.  **Left Lower Lung (Outer Basal Segment):** A small round area, about 5mm in size, is seen near the lining of the lung. This area looks clear, and there are patches of higher density around it, which could be related to previous inflammation. Follow-up imaging is recommended.  **Mediastinum:** The middle area of the chest is normal, and the trachea (windpipe) and major bronchi (air passages) are open with no blockages.  Heart: The heart's size and shape are normal with no signs of abnormalities.  **Aorta:** A small area with increased density is seen on the wall of the aorta (main artery), which is consistent with mild hardening of the artery (atherosclerosis).  **Pleural Effusion:** No fluid is seen in the spaces surrounding the lungs.  **Chest Wall:** The bones of the chest are intact, and the chest walls are symmetrical with no signs of injury.  **Liver:** A small low-density area is observed in the left side of the liver, approximately 20mm x 20mm in size. It is recommended to perform an abdominal scan for further assessment. |
| **Conclusion (Based on 2023-09-18 CT with Contrast):**  **Right Upper Lobe Nodule:** The nodule has increased in size compared to the previous scan, and is suspicious for a new growth. Further clinical and pathological examination is recommended.  **Bilateral Lungs:** Scattered chronic inflammation and fibrotic lesions are present, with changes in the right upper lobe and bilateral lower lobes similar to the previous scan. Follow-up with clinical monitoring is recommended.  **Left Lung Oblique Fissure Small Nodule:** Follow-up CT is recommended.  **Left Lower Lobe Outer Basal Segment Cystic Lesion:** Follow-up CT is recommended.  **Aortic Atherosclerosis:** No significant change.  **Liver Left Lobe Hypodensity:** A hypodense lesion is observed, measuring approximately 20mm x 20mm. Further abdominal examination is recommended. | **Conclusion:**  **Right Upper Lobe Nodule:** The nodule has grown since the last scan, and it may be a new tumor. A biopsy or additional tests are recommended to confirm.  **Bilateral Lungs:** There are signs of chronic inflammation and scarring in both lungs, which have remained stable since the last scan. Regular follow-up is recommended.  **Left Lung Oblique Fissure Small Nodule:** A small nodule is present in the left lung. It is recommended to monitor it with follow-up imaging.  **Left Lower Lobe Outer Basal Segment Cystic Lesion:** A small cystic lesion is observed in the lower part of the left lung. It should be checked in future scans.  **Aortic Atherosclerosis:** No significant changes in the aorta compared to previous scans, but it is important to monitor for cardiovascular health.  **Liver Left Lobe Hypodensity:** A small low-density area is noted in the liver, and additional abdominal tests are suggested for further evaluation. |
