# Supplementary material for: Enhancing Physician-Patient Communication in Oncology Using GPT-4 Through Simplified Radiology Reports: Multicenter Quantitative Study
Source: J Med Internet Res. 2025 Apr 17;27:e63786. doi: 10.2196/63786 (PMC12046253; doi:10.2196/63786)
Supplement: Multimedia Appendix 2 [file jmir_v27i1e63786_app2.docx]

| **Table S2**. Demographic characteristics of volunteers. | |
| --- | --- |
| Demographic Characteristic | Volunteers (N = 30) |
| Age (years) |  |
| Mean Range | 43.5 (20–67) |
| Sex |  |
| Man | 15 |
| Woman | 15 |
| Educational Background |  |
| Junior High School | 6 |
| High School | 12 |
| **Bachelor's Degree** | 9 |
| Master's Degree | 3 |
| Non-Medical Background (%) | 100% |
