# Supplementary material for: Enhancing Physician-Patient Communication in Oncology Using GPT-4 Through Simplified Radiology Reports: Multicenter Quantitative Study
Source: J Med Internet Res. 2025 Apr 17;27:e63786. doi: 10.2196/63786 (PMC12046253; doi:10.2196/63786)
Supplement: Multimedia Appendix 3 [file jmir_v27i1e63786_app3.docx]

| **Table S3.** CRIE 3.0 Readability Metrics Comparison: Original Radiology Reports (ORRs) vs. Interpretative Radiology Reports (IRRs). | | | | | |
| --- | --- | --- | --- | --- | --- |
| **Category** | **Metric** | **ORRs [95%CI]** | **IRRs [95%CI]** | **Range and Grading (Based on CRIE 3.0)** | **Explanation** |
| Vocabulary | Advanced Vocabulary Ratio (%) | 18.5 [17.8, 19.2] | 3.2 [2.9, 3.5] | Low: 0-5%, Moderate: 5-15%, High: >15% | Indicates the percentage of words that are considered advanced. A higher value in the original report suggests more challenging vocabulary, making it harder for general audiences to understand. |
|  | Lexical Diversity Index (TTR) | 0.75 [0.72, 0.78] | 0.45 [0.42, 0.48] | Low: <0.3, Moderate: 0.3-0.6, High: >0.6 | Represents the diversity of words used in the text. The original report has a higher diversity of terms, indicating a rich but complex vocabulary, while the interpreted report is more straightforward. |
| Sentence | Average Sentence Length (words) | 32 [30.5, 33.5] | 18 [17, 19] | Short: <10 words, Moderate: 10-20 words, Long: >20 words | Reflects the average number of words per sentence. The original report has longer sentences, making it more difficult to read compared to the interpreted report, which has moderate-length sentences. |
|  | Complex Sentence Ratio (%) | 45 [42, 47] | 12 [10, 14] | Low: 0-15%, Moderate: 15-30%, High: >30% | The percentage of sentences that are complex (having multiple clauses). The original report has a high complexity ratio, whereas the interpreted report is simpler and more accessible. |
| Discourse | Coherence Score | 70 [68, 72] | 85 [83, 87] | Low: <60, Moderate: 60-80, High: >80 | Measures how logically connected the text is, on a scale of 0 to 100. The interpreted report has a higher coherence score, indicating that it is easier to follow. |
|  | Average Paragraph Length (words) | 220 [210, 230] | 140 [130, 150] | Short: <80 words, Moderate: 80-150 words, Long: >150 words | Indicates the average length of paragraphs. The original report contains longer paragraphs, which can be more challenging to read, while the interpreted report has moderately long paragraphs. |
| Cognitive Load | Information Density Index | 0.72 [0.70, 0.74] | 0.45 [0.43, 0.47] | Low: <0.3, Moderate: 0.3-0.6, High: >0.6 | Reflects the amount of new information per unit of text. The original report has a higher information density, making it more challenging, while the interpreted report is less dense and easier to digest. |
|  | Complex Vocabulary Density (%) | 18.5 [17.5, 19.5] | 4 [3.5, 4.5] | Low: 0-5%, Moderate: 5-15%, High: >15% | Indicates the percentage of words that are less common and harder to understand. The original report has a high density of complex vocabulary, while the interpreted report is more reader-friendly. |
