# Supplementary material for: Enhancing Physician-Patient Communication in Oncology Using GPT-4 Through Simplified Radiology Reports: Multicenter Quantitative Study
Source: J Med Internet Res. 2025 Apr 17;27:e63786. doi: 10.2196/63786 (PMC12046253; doi:10.2196/63786)
Supplement: Multimedia Appendix 4 [file jmir_v27i1e63786_app4.docx]

| **Table S4.** Cohen's Kappa for Categorical Evaluations of Comprehension Dimensions. | | | |
| --- | --- | --- | --- |
| Comprehension Dimension | Cohen's Kappa Value | *P*-value | Agreement Level |
| Understanding of Report Structure | 0.76 | 0.002 | Substantial |
| Understanding of Professional Terminology | 0.80 | < .001 | Substantial |
| Interpretation of Imaging Results | 0.82 | < .001 | Substantial |
| Understanding of Report Conclusion | 0.85 | < .001 | Almost Perfect |
| Overall Understanding and Application | 0.79 | 0.003 | Substantial |
