# Supplementary material for: Enhancing Physician-Patient Communication in Oncology Using GPT-4 Through Simplified Radiology Reports: Multicenter Quantitative Study
Source: J Med Internet Res. 2025 Apr 17;27:e63786. doi: 10.2196/63786 (PMC12046253; doi:10.2196/63786)
Supplement: Multimedia Appendix 5 [file jmir_v27i1e63786_app5.docx]

| **Table S5**. Intraclass Correlation Coefficients (ICCs) for Continuous Evaluations of Comprehension Dimensions. | | | |
| --- | --- | --- | --- |
| Comprehension Dimension | ICC Value | *P*-value | Agreement Level |
| Understanding of Report Structure | 0.82 | < .001 | Excellent |
| Understanding of Professional Terminology | 0.85 | < .001 | Excellent |
| Interpretation of Imaging Results | 0.87 | < .001 | Excellent |
| Understanding of Report Conclusion | 0.91 | < .001 | Excellent |
| Overall Understanding and Application | 0.84 | < .001 | Excellent |
