# Supplementary material for: Enhancing Physician-Patient Communication in Oncology Using GPT-4 Through Simplified Radiology Reports: Multicenter Quantitative Study
Source: J Med Internet Res. 2025 Apr 17;27:e63786. doi: 10.2196/63786 (PMC12046253; doi:10.2196/63786)
Supplement: Multimedia Appendix 6 [file jmir_v27i1e63786_app6.docx]

**Multimedia Appendix 1: Reconstructed ChatGPT Interaction Examples**

In accordance with the JMIR Publications policy on AI usage [1], this appendix provides **representative examples of GPT-4 (OpenAI)** interactions that contributed to the preparation of this manuscript. GPT-4 was used for language refinement, paragraph organization, and clarity improvement. **All content was reviewed, revised, and validated by human authors.**

⚠️ Due to platform limitations, **complete transcripts are no longer available**. However, the following examples have been **reconstructed** based on the comparison of manuscript versions between June 20, 2024 (first draft), and March 20, 2025 (final submission). These reconstructions are the only available records of ChatGPT use for this work.

All GPT-4 outputs are displayed in textboxes to distinguish them clearly. Authors did not rely on GPT-generated references or unverified facts; all data interpretation and scientific statements were independently verified.

*For more information on editorial policy, see: [Best Practices for Using AI Tools as an Author](https://www.jmir.org/2023/1/e51584).*

---

**Example 1: Abstract Refinement**

**Date:** 2024-06-22

**User Prompt:** Please polish the following abstract to make it more formal and suitable for a medical journal.

**Original Text:**

"Radiology reports often contain complex medical terms that patients cannot understand well. We used GPT-4 to make them simpler and found that patient comprehension improved."

**ChatGPT Output:**

"Radiology reports frequently include complex medical terminology that may hinder patient comprehension. In this study, we employed GPT-4 to simplify radiology reports and observed significant improvements in patient understanding."

**Final Adopted Version:**

"These reports are often filled with technical jargon, making them challenging for patients to understand and affecting their engagement and decision-making."

---

**Example 2: Introduction Enhancement**

**Date:** 2024-06-24

**User Prompt:** Improve the paragraph to include more background about how LLMs can improve radiology report readability and their limitations.

**Original Text:**

"LLMs have been shown to simplify reports, but they haven’t been used much in clinical settings."

**ChatGPT Output:**

"Previous studies have demonstrated that large language models (LLMs) can effectively process and simplify complex medical texts, showing considerable potential in enhancing accessibility and comprehension for patients. However, their application in real-world clinical workflows, particularly regarding physician-patient communication, remains underexplored."

**Final Adopted Version:**

"Previous studies have demonstrated that large language models (LLMs) can effectively process and simplify complex medical texts, showing considerable potential in enhancing accessibility and comprehension for patients..."

---

**Example 3: Methods Section Clarity**

**Date:** 2024-06-28

**User Prompt:** Please clarify this paragraph about the prompt design.

**Original Text:**

"We wrote a prompt and input the ORR to GPT-4. It returned a new report that is simpler."

**ChatGPT Output:**

"To generate Interpretive Radiology Reports (IRRs), we used a standardized and structured prompt template. The primary goal of this prompt was to simplify the technical language of the reports while maintaining clinical accuracy and ensuring accessibility for non-medical readers."

**Final Adopted Version:**

"To generate Interpretive Radiology Reports (IRRs), we used a standardized and structured prompt template. The primary goal of this prompt was to simplify the technical language of the reports while maintaining clinical accuracy and ensuring accessibility for non-medical readers."

---

**Example 4: Statistical Description Adjustment**

**Date:** 2025-01-18

**User Prompt:** Can you rewrite this sentence using proper statistical reporting style?

**Original Text:**

"The difference is significant. Patients took less time to read IRRs."

**ChatGPT Output:**

"The average reading time for IRRs (589.92 seconds) was significantly shorter than that for ORRs (674.86 seconds), with a P value < .001."

**Final Adopted Version:**

"The average reading time for IRRs was 589.92 seconds, significantly shorter than the 674.86 seconds observed for ORRs (P < .001)."

---

**Example 5: Ethical Considerations Language Polishing**

**Date:** 2025-02-03

**User Prompt:** Please polish the following ethical discussion.

**Original Text:**

"AI needs to be carefully supervised. There are issues like legal responsibility and bias."

**ChatGPT Output:**

"The integration of LLMs in healthcare introduces important ethical and legal considerations. Issues surrounding patient data privacy, AI bias, and the legal implications of AI-generated medical reports must be addressed."

**Final Adopted Version:**

"The integration of LLMs in healthcare introduces important ethical and legal considerations. Issues surrounding patient data privacy, AI bias, and the legal implications of AI-generated medical reports must be addressed."

---

This appendix illustrates the types of language, clarity, and consistency improvements provided by GPT-4. All outputs were verified and revised as necessary by the authors prior to inclusion in the manuscript.

Note: Figure and table generation, data analysis, and result tabulation were performed manually and are not included in the AI-assisted components described here.
